# DOMINO-AD protocol: donepezil and memantine in moderate to severe Alzheimer's disease – a multicentre RCT

**DOI:** 10.1186/1745-6215-10-57

**Published:** 2009-07-24

**Authors:** Rob Jones, Bart Sheehan, Patrick Phillips, Ed Juszczak, Jessica Adams, Ashley Baldwin, Clive Ballard, Sube Banerjee, Bob Barber, Peter Bentham, Richard Brown, Alistair Burns, Tom Dening, David Findlay, Richard Gray, Mary Griffin, Clive Holmes, Alan Hughes, Robin Jacoby, Tony Johnson, Roy Jones, Martin Knapp, James Lindesay, Ian McKeith, Rupert McShane, Ajay Macharouthu, John O'Brien, Caroline Onions, Peter Passmore, James Raftery, Craig Ritchie, Rob Howard

**Affiliations:** 1 Section of Old Age Psychiatry, The University of Nottingham, A Floor, South Block, Queen's Medical Centre, Nottingham NG7 2UH, UK; 2 Health Sciences Research Institute, Warwick Medical School, University of Warwick, Coventry CV4 7AL, UK; 3 MRC Clinical Trials Unit, 22 Euston Road, London NW1 2DA, UK; 4 Head of NHS Statistical Support Team, Centre for Statistics in Medicine, Wolfson College Annexe, University of Oxford, Linton Road, Oxford OX2 6UD, UK; 5 PO70, Institute of Psychiatry, De Crespigny Park, London SE5, UK; 6 Knowlsey Resource & Recover Centre, Whiston Hospital, Warrington Road, Prescot, Merseyside L35 5DR, UK; 7 Wolfson Centre for Age Related Disease, Guy's Campus, King's College, London SE1 1UL, UK; 8 PO26, Section of Mental Health and Ageing, Health Services Research Department, The David Goldberg Centre, The Institute of Psychiatry, De Crespigny Park, London SE5 8AF, UK; 9 Institute for Ageing and Health, University of Newcastle, Wolfson Research Centre, Newcastle General Hospital, Westgate Road, Newcastle-upon-Tyne, NE4 6BE, UK; 10 Queen Elizabeth Psychiatric Hospital, Mindelsohn Way, Edgbaston, Birmingham B15 2QZ, UK; 11 Department of Psychiatry, Kings College London, Institute of Psychiatry, De Crespigny Park, London SE5 8AF, UK; 12 PBS 18, 2^nd ^Floor, Education and Research Centre, School of Psychiatry and Behavioural Sciences, Wythenshawe Hospital, Southmoor Road, Manchester M23 9LT, UK; 13 Older People's Mental Health Service, Box 311, Fulbourn Hospital, Cambridge CB1 5EF, UK; 14 Dundee Community Health Partnership, Gowrie House, Royal Dundee Liff Hospital, Dundee DD2 5NF, UK; 15 University of Birmingham, Park Grange, 1 Somerset Road, Birmingham B15 2RR, UK; 16 PO70 Institute of Psychiatry, De Crespigny Park, London SE5, UK; 17 Department of Old Age Psychiatry, Moorgreen Hospital, Botley Road, West End, Southampton, Hants SO30 3JB, UK; 18 Department of Geriatric Psychiatry, Inverclyde Royal Hospital, Larkfield Road, Inverclyde PA16 0NX, UK; 19 University of Oxford, Department of Psychiatry, The Warneford Hospital, Oxford OX3 7JX, UK; 20 MRC Biostatistics Unit (& MRC Clinical Trials Unit, London), Institute of Public Health, University Forvie Site, Robinson Way, Cambridge CB2 2SR, UK; 21 Research Institute for Care of the Elderly, St Martin's Hospital, Bath BA2 5RP, UK; 22 Department of Economics of Mental Health, Kings College London, Institute of Psychiatry, De Crespigny Park, London SE5 8AF, UK; 23 Department of Health Sciences, University of Leicester, Leicester General Hospital, Leicester LE5 4PW, UK; 24 Old Age Psychiatry, University of Newcastle, Wolfson Research Centre, Newcastle General Hospital, Westgate Road, Newcastle-upon-Tyne, NE4 6BE, UK; 25 The Fulbrook Centre, The Churchill Hospital, Oxford OX3 7JU, UK; 26 North West Kilmarnock Area centre, Western Road, Kilmarnock KA13 1NQ, UK; 27 Wolfson Research Centre, Newcastle General Hospital, Westgate Road, Newcastle-upon-Tyne NE4 6BE, UK; 28 PO70 Institute of Psychiatry, De Crespigny Park, London SE5, UK; 29 Whitla Medical Building, 97 Lisburn Road, Belfast BT9 7BL, UK; 30 School of Medicine, University of Southampton, Mail point 728, Bolrewood, Bassett Crescent, East Southampton SO16 7PX, UK; 31 Charing Cross Hospital, Claybrook Centre, 37 Claybrook Road, London W6 8LN, UK; 32 PO70, Institute of Psychiatry, De Crespigny, London SE5 8AF, UK

## Abstract

**Background:**

Alzheimer's disease (AD) is the commonest cause of dementia. Cholinesterase inhibitors, such as donepezil, are the drug class with the best evidence of efficacy, licensed for mild to moderate AD, while the glutamate antagonist memantine has been widely prescribed, often in the later stages of AD. Memantine is licensed for moderate to severe dementia in AD but is not recommended by the England and Wales National Institute for Health and Clinical Excellence. However, there is little evidence to guide clinicians as to what to prescribe as AD advances; in particular, what to do as the condition progresses from moderate to severe. Options include continuing cholinesterase inhibitors irrespective of decline, adding memantine to cholinesterase inhibitors, or prescribing memantine instead of cholinesterase inhibitors. The aim of this trial is to establish the most effective drug option for people with AD who are progressing from moderate to severe dementia despite treatment with donepezil.

**Method:**

DOMINO-AD is a pragmatic, 15 centre, double-blind, randomized, placebo controlled trial. Patients with AD, currently living at home, receiving donepezil 10 mg daily, and with Standardized Mini-Mental State Examination (SMMSE) scores between 5 and 13 are being recruited. Each is randomized to one of four treatment options: continuation of donepezil with memantine placebo added; switch to memantine with donepezil placebo added; donepezil and memantine together; or donepezil placebo with memantine placebo. 800 participants are being recruited and treatment continues for one year. Primary outcome measures are cognition (SMMSE) and activities of daily living (Bristol Activities of Daily Living Scale). Secondary outcomes are non-cognitive dementia symptoms (Neuropsychiatric Inventory), health related quality of life (EQ-5D and DEMQOL-proxy), carer burden (General Health Questionnaire-12), cost effectiveness (using Client Service Receipt Inventory) and institutionalization. These outcomes are assessed at baseline, 6, 18, 30 and 52 weeks. All participants will be subsequently followed for 3 years by telephone interview to record institutionalization.

**Discussion:**

There is considerable debate about the clinical and cost effectiveness of anti-dementia drugs. DOMINO-AD seeks to provide clear evidence on the best treatment strategies for those managing patients at a particularly important clinical transition point.

**Trial registration:**

Current controlled trials ISRCTN49545035

## Background

Acetylcholinesterase inhibitors are widely prescribed to patients with mild to moderate Alzheimer's disease (AD), with studies showing they improve cognition and stabilize cognition, function and behaviour for up to 6–12 months (see below). Although there is evidence that these drugs are effective in the mild to moderate range of severity, there is at present little evidence to guide clinicians at the critical decision point when patients deteriorate beyond moderate to severe dementia. Memantine has systematic review level evidence to support efficacy in late stage AD [1] and has been widely prescribed at this stage. Again, however, there is not an adequate evidence base to guide decisions about patients compliant with a cholinesterase inhibitor but who have reached the moderate to severe point where they might be considered for memantine treatment.

There is therefore a pressing clinical need to provide an evidence base for physicians on which to base decisions about continued prescribing of cholinesterase inhibitors in patients as they reach the moderate to severe stage of AD but there have been no clinical trials that can provide this evidence. There is also a need to evaluate the use of memantine, which already has a licence for the treatment of moderate to severe AD, alone and in combination with a cholinesterase inhibitor at the point where patients make the transition to moderate to severe disease. To inform clinical practice and relevant decision-making bodies (such as, for England and Wales, the National Institute for Health and Clinical Excellence, NICE) most effectively, any trials that examine these issues should be pragmatic and based on a representative patient population in whom clinicians currently have genuine uncertainty about how to proceed with treatment. Important outcome measures for such a trial should include preservation of activities of daily living and independence as well as cost-utility data.

By recruiting patients who have reached the transition point between moderate and severe dementia, and who are already receiving anticholinesterase treatment, DOMINO-AD is designed to answer clinical questions about efficacy and cost-effectiveness that have real relevance to clinicians and policy making organisations.

### Clinical Studies

A number of studies have demonstrated that acetylcholinesterase inhibitors modestly improve cognition in a subgroup of patients with mild to moderate AD and stabilize cognition, function and behaviour for 6 months [2-11] and may continue to exert benefit for 12 months [12,13]. Evidence has also been presented to suggest that these drugs may continue to have a beneficial effect for up to 2 years [14] although a recent influential systematic review concluded that benefits of treatment are modest and that the methodological quality of most published trials could be questioned [15].

Acetylcholine and choline acetyltransferase levels do not begin to fall significantly until dementia is advanced [16,17]. Hence, there would be good theoretical reasons to anticipate a therapeutic effect of cholinesterase inhibition at later stages of disease than the currently licensed indication of mild to moderate severity. Trial evidence for this comes from four particular sources.

Firstly, randomized double-blind placebo-controlled trials of cholinesterase inhibitors that have included moderate to severely affected patients [18] have shown significant benefits over 24 weeks in cognitive, behavioural and functional outcomes in a group of patients whose SMMSE scores ranged from 5–17 (mean score for patients receiving donepezil = 11.7).

Secondly, secondary analyses of trial data from mild to moderate patients examining the subgroup of patients with more severe illness have also shown benefits. Wilkinson [19] performed a post hoc analysis on pooled data from 124 patients with SMMSE scores of 10–12 from the 4 pivotal galantamine studies. Cognitive and functional abilities were significantly improved in galantamine treated participants. Burns [20] retrospectively analyzed pooled data from 117 patients selected from three RCTs of rivastigmine on the basis of a SMMSE score of 10–12 points. Rivastigmine treatment over 6 months showed significant benefits in cognitive and behavioural domains. Further, Gauthier [21] and Feldman [22] reanalyzed earlier Feldman [18] data focussing on 145 patients with a SMMSE score of 5–12. At week 24, using the last observation carried forward principle for imputing missing data, the mean differences in mean change from baseline scores were 2.0 points for the SMMSE and 7.4 points for the Severe Impairment Battery (SIB) in favour of the donepezil treated participants and Clinician's Interview-Based Impression of Change-Plus (CIBIC-plus) scores were significantly improved compared with placebo with a 0.70 point mean treatment difference.

Thirdly, long term trial data within which participants have progressed to more severe disease stages of illness can also demonstrate apparent continued efficacy in moderate to severe patients. In the AD2000 trial [14] 49% of participants randomized to treatment with donepezil had SMMSE scores of 10–18 points at study entry. Over the 2-year study period, the donepezil group averaged SMMSE scores 0.8 points higher than the placebo group. This benefit was not restricted to any subgroup of patients in terms of severity rating and was maintained over the trial period. Raskind [23] demonstrated benefits of continuing galantamine treatment for 36 months in a group of patients whose mean SMMSE score at entry had been 19.7 points and a proportion of whom would have entered the moderate to severe category during the course of the study.

Fourthly, trials examining the effects of cholinesterase inhibitor withdrawal provide some information. Typically, following a placebo washout period of 6 weeks at the end of a trial, the benefits of 24 weeks of donepezil treatment in terms of cognition and global function are lost [24]. Most of such washouts have been at the end of relatively short trials at which point patients have still been only mildly to moderately affected. There are no published randomized controlled trials examining the effects of treatment withdrawal in patients at the moderate to severe boundary that would help clinicians to make decisions at the point where NICE guidance advises stopping. Holmes [25] showed behavioural benefits of continuing donepezil in a group of patients selected on the basis of marked neuropsychiatric symptoms (NPI score>11) at baseline and with a mean SMMSE score of 21.1 points.

Additionally, there is intriguing preliminary evidence that a combination of a cholinesterase inhibitor and memantine might be particularly beneficial in patients at this severity point. For example, a study of 404 patients with moderate to severe AD (SMMSE 5–14) who had been stabilized on donepezil treatment for at least 6 months, investigated the effect of adding memantine 10 mg b.d. for 6 months [26]. The change in total mean (standard error) scores favoured memantine over placebo for the SIB; 1.0 (0.7) vs. -2.4 (0.74), Alzheimer's Disease Cooperative Study Activities of Daily Living 19 (ADCS-ADL 19); -1.7 (0.51) vs. -3.3 (0.55) and CIBIC-Plus; 4.38 (0.081) vs. 4.64 (0.087). All other secondary measures showed significant benefits of memantine treatment. Treatment discontinuations because of adverse events were seen in 7.4% receiving memantine and 12.4% in those receiving placebo. An unpublished Phase III study in mild to moderate AD patients, however, has suggested that combinations of a variety of cholinesterase inhibitors and memantine may not provide additional benefits over monotherapy in this less impaired group [27]. Overall, the preliminary and post-hoc evidence of an effect on behaviour has not yet been backed up by RCT evidence which tests this question [28,29] and there is a clear need for more data.

### NICE Guidance

NICE recommended in 2001 [30] that cholinesterase inhibitors (donepezil, rivastigmine and galantamine) should be offered to patients with mild to moderate AD whose MMSE score was above 12 points. NICE guidance from 2001–2005 was that prescription should only be continued while the MMSE score remained above 12 points and the patient's global, functional and behavioural condition remained at a level where the drug was considered to have a worthwhile effect. This recommendation was made on the grounds of cost-containment, rather than clinical efficacy, since many of the patients who entered the trials that established efficacy had MMSE scores of as low as 10 points.

Current NICE guidance for England and Wales [31], which although amended in response to judicial review since its original publication remains substantially the same in its effect, recommends that the three acetylcholinesterase inhibitors donepezil, galantamine and rivastigmine are used as options in the management of people with AD of moderate severity (MMSE score of between 10 and 20 points). Patients continuing on the drugs should be reviewed six monthly by MMSE score and global, functional and behavioural assessment, incorporating the views of carers. Drug treatment should only be continued while patients' MMSE score remains above 10 points and their global, functional and behavioural condition remains at a level where the drug is considered to be having a worthwhile effect. Memantine is not recommended as a treatment option for people with moderately severe to severe AD except as part of a clinical study.

### Aim of the study

The aim of the DOMINO-AD study is to determine, in a factorial (2 × 2) design, whether there is worthwhile benefit for patients, for whom there is uncertainty on whether or not to continue cholinesterase inhibitors, from: 1) adding memantine to donepezil, 2) switching to memantine or 3) continuing donepezil compared to 4) placebo.

### Objectives

#### Primary Objectives

The trial will test a number of hypotheses in memory clinic patients who have declined in terms of cognitive function to reach the transition point to moderate-to-severe AD. These hypotheses are:-

a) patients with AD who continue donepezil beyond the moderate to severe transition point will show a significantly smaller decline on ratings of cognitive function and activities of daily living over the following 12 months than those discontinuing donepezil, with analysis using all trial participants;

b) patients with AD who commence memantine therapy at the moderate to severe transition point will show a significantly smaller decline on ratings of cognitive function and activities of daily living over the following 12 months than those who do not, with analysis using all trial participants;

c) patients given the combination of memantine and donepezil at the moderate to severe transition point will show additive or synergistic significant benefits on measures of activities of daily living and cognitive function after 12 months compared to those patients continuing on either monotherapy.

#### Secondary Objectives

Secondary hypotheses to be tested are:-

a) patients with AD who continue donepezil beyond the moderate to severe transition point will show a significantly smaller deterioration on ratings of non-cognitive symptoms and health related quality of life over the following 12 months than those discontinuing donepezil, with analysis using all trial participants;

b) patients with AD who commence memantine therapy at the moderate to severe transition point will show a significantly smaller deterioration on ratings of non-cognitive symptoms and health related quality of life over the following 12 months than those who do not, with analysis using all trial participants;

c) patients given the combination of memantine and donepezil at the moderate to severe transition point will show additive or synergistic significant benefits on measures of non-cognitive symptoms and health related quality of life after 12 months compared to those patients continuing on either monotherapy;

d) treatment of patients with donepezil beyond the moderate to severe transition point will be more cost-effective than discontinuing donepezil; memantine therapy will be more cost-effective than placebo; the combination of memantine and donepezil will be more cost-effective than monotherapy;

e) patients who continue on donepezil beyond the moderate to severe transition point will be institutionalized later than those who do not; patients who commence memantine therapy will be institutionalized later than those taking placebo; patients who commence the combination of memantine and donepezil will be institutionalized later than those on monotherapy.

For carers, parallel secondary objectives concern changes in psychological morbidity and health related quality of life.

## Methods

### Design

**Figure 1:**
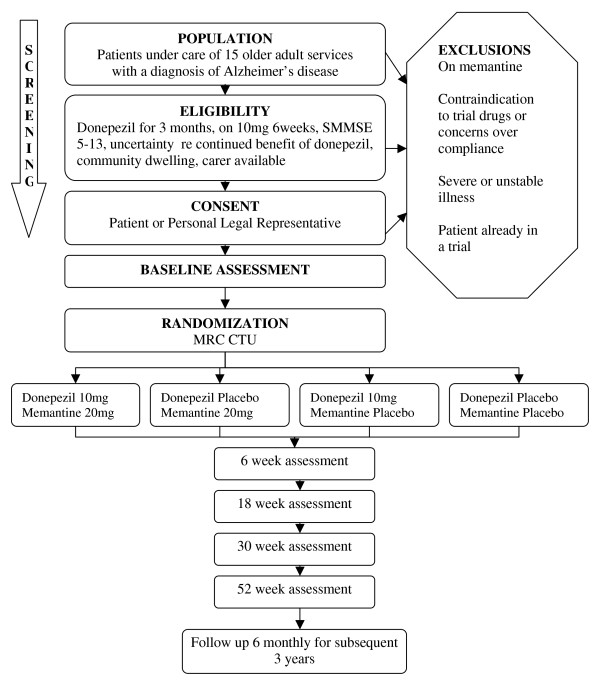
**Trial Flow Chart**.

This is a pragmatic, multicentre, double-blind (with patient, carer, clinician, outcome assessor and investigators blinded), randomized, placebo controlled (double dummy), parallel group, 2 × 2 factorial clinical trial. Figure 1 illustrates the trial design. All participants receive trial interventions for 52 weeks. Participants are randomized to one of four arms centrally by the Medical Research Council (MRC) Clinical Trials Unit in London via telephone. Randomization is by dynamic allocation using minimization to ensure balanced allocation across the following factors: centre, duration of donepezil treatment prior to randomization, baseline SMMSE score and age.

#### (Arm 1) Combination of donepezil plus memantine

Participants in this arm continue with their current donepezil 10 mg/day regimen and immediately commence active memantine at a dose of 5 mg per day, increasing in 5 mg increments weekly until 20 mg per day is achieved from week 4 onwards.

#### (Arm 2) Withdrawal of donepezil and prescription of memantine

Participants in this arm immediately commence active memantine at a dose of 5 mg per day, increasing in 5 mg increments weekly until 20 mg per day is achieved from week 4 onwards. The donepezil dose is reduced to 5 mg daily in weeks 1 to 4 and replaced with placebo donepezil in week 5.

#### (Arm 3) Continued prescription of donepezil monotherapy

Participants in this arm continue with their current donepezil 10 mg/day regimen and immediately commence placebo memantine.

#### (Arm 4) Withdrawal of donepezil

Participants in this arm immediately commence placebo memantine dose escalation and switch to donepezil 5 mg daily in weeks 1 to 4, which is replaced with placebo donepezil in week 5.

### Planned inclusion/exclusion criteria

#### Inclusion criteria

People are eligible to participate if they are patients who meet standardized clinical McKhann criteria [32] for probable or possible AD, have been continuously prescribed donepezil for at least 3 months and continuously prescribed 10 mg donepezil for the previous 6 weeks. They must have had no changes in prescription of any psychotropic drugs (antipsychotic, antidepressant, benzodiazepine) in the previous 6 weeks, the prescribing clinician must consider (based on NICE guidance, discussions with patient and carer and clinical judgement) that change of drug treatment (i.e. stop donepezil or introduce memantine) may be appropriate and on testing with the SMMSE, the standardized assessment of cognitive function, the score is between 5 and 13. Also, to be eligible, the participant must be community resident with a family or professional carer or be visited on at least a daily basis by a carer. Participants must have agreed to take part if considered capable and the main carer (informal or professional) must also have given consent to his/her own involvement and to the participant's involvement.

#### Exclusion criteria

These include severe, unstable or poorly controlled medical conditions apparent from physical examination or clinical history, current prescription of memantine, contra-indications or previous adverse or allergic reactions to trial drugs, involvement in another clinical trial or that the clinician considers the patient would not be compliant.

### Recruitment/consent procedures

Participants are identified from patients with AD meeting study eligibility criteria who are being followed up in memory clinics, out patient clinics or other components of specialist mental health, geriatric medicine or neurology services. Once a potential participant has been identified, the clinician obtains verbal consent to pass his/her details to the research worker who then recruits the patient in line with the trial standard operating procedures.

Where possible, fully informed consent is obtained from the patient. However, the majority of patients with moderate to severe dementia lack the necessary mental capacity to give fully informed consent. In this situation, agreement to participate in the study is still obtained to the patient's best level of understanding and the patient is not enrolled if they refuse or show significant distress. For sites in England and Northern Ireland, in line with The Medicines for Human Use (Clinical Trials) Regulations 2004, consent is also obtained from the patient's personal legal representative. For sites in Scotland, in line with the Adults with Incapacity (Scotland) Act 2000, consent is obtained from the adult's proxy. If neither is available to consent, the patient is ineligible for the study.

### Assessments

Study assessment measures will be applied at baseline prior to randomization, at week 6 to assess the acute effects of donepezil withdrawal, at week 18, week 30 and at week 52. Participants will then be followed up every 26 weeks for 3 years by telephone interview to establish whether and on what date they entered a care institution. Trial completion is defined as completion of 52 weeks on the trial medication or discontinuation of follow-up for any cause. Participants who discontinue taking the trial medication are encouraged to remain in follow-up. Participants may not formally discontinue their follow-up and remain on the trial medication. Arrangements for continued provision of the trial medication at the end of the trial will be made on an individual basis by the clinician responsible for the participant's care.

#### Primary Outcomes Measures

##### Standardized Mini-Mental State Examination (SMMSE)

The Mini-Mental State Examination is a well-established measure of cognitive function in elderly people. It shows good test-retest and inter-rater reliability and performs satisfactorily against more detailed measures of cognitive function [33]. The Standardized Mini-Mental State Examination (SMMSE) has been developed to improve the reliability of the original instrument [34] and will be used to assess decline in cognitive function during the trial. Scores range from 30 (unimpaired) to 0 (impaired).

##### Bristol Activities of Daily Living Scale (BADLS)

The BADLS was specifically designed for use with dementia patients living in the community and participating in clinical trials [35]. The BADLS is sensitive to change, correlates well with economic outcomes and, despite being a carer rated instrument, appears to have good test-retest reliability, on a stringent measure, and, additionally, the levels of disability between which the scale aims to discriminate were also carer generated, giving some perspective on the value of change. The BADLS will be used to assess activities of daily living during the trial. Scores range from 0 (unimpaired) to 60 (impaired).

#### Secondary Outcome Measures

##### Client Service Receipt Inventory (CSRI)

The CSRI describes service use, informal care and other aspects of accommodation and care pertinent to the costing of interventions and their implications [36]. Parallel sections of the CSRI will be used for those in: residential care, at home with co-resident carer, and at home without co-resident carer.

##### EuroQol EQ-5D (EQ-5D)

The EQ-5D instrument is a generic, utility-based health related quality of life (HRQoL) measure [37]. It can be simply administered to patients or carers in the form of a self-completed questionnaire and has been used in patients with neurological disorders. In this trial, the carer will be completing the questionnaire. There are two core components to the instrument: a description of the respondent's own health using a health state classification system with five dimensions, and a rating on a visual analogue thermometer scale.

##### DEMQOL-Proxy

DEMQOL-Proxy is a 31 item, disease specific instrument for evaluating HRQoL in dementia, which shows comparable psychometric properties to the best available instruments and has been validated in a UK population [38], and given doubts about how well the EQ-5D performs with people with dementia this usefully complements it.

##### Neuropsychiatric Inventory (NPI)

The NPI assesses twelve domains of possible behavioural disturbance in dementia – using a screening strategy to save time [39]. The NPI will be used in DOMINO-AD to measure the caregiver's assessment of nature, frequency and severity of Behavioural Psychological Symptoms of Dementia (BPSD). NPI scores range from 0 (no disturbance) to 144 (maximum disturbance).

##### General Health Questionnaire 12 (GHQ-12)

The GHQ-12 is a well validated, widely used, self-rated instrument for detecting psychological morbidity and psychiatric disorder [40]. In DOMINO-AD GHQ-12 will be used to measure levels of psychological distress in the carers of study patients. Scores range from 0 (not distressed) to 12 (distressed) using the GHQ scoring method, with a cut off of 2/3 utilised to discriminate cases from non-cases.

##### Institutionalization

This will be assessed via a simple question as to where the patient is living.

### Statistics

#### Sample Size Calculation

The trial aims to recruit 800 patients over a period of 2 years. Even allowing for a 25% loss to follow-up, this sample size will provide over 90% power to detect small (0.25 standard deviations) overall treatment effects on the primary outcome measures with 90% power at p < 0.01. It will also be sufficient to detect small differences (0.25 standard deviations) in the primary outcome measures at any one assessment point with 80% power at p < 0.05, and small to moderate differences (0.3 standard deviations) with 90% power at p < 0.05. These differences are equivalent to 1–2 point improvements in the SMMSE and BADLS, which are considered the minimal clinically relevant differences. The sample size calculations assume a correlation between serial measurements of the SMMSE/BADLS of around 0.6 (multiplying factor of 0.34 based on 1 baseline and 4 post-randomization measurements, as described by Machin et al [41]) anticipating an analysis of covariance with repeated measures.

#### Analyses

The primary analyses of the effect of donepezil and memantine on BADLS and SMMSE will be analyzed using multilevel modelling repeated measures (MMRM) regression methods, adjusted for baseline scores. This approach gives greater power to detect differences than simple t-tests on differences and will allow investigations to be made to determine how long any benefits of treatment persist, and whether the mode of action is one of symptomatic relief or disease modification. By including all time points, MMRM analyses minimise the effect of any missing data. Nevertheless, vigorous efforts will be made to minimise missing data, and sensitivity analyses will be undertaken to explore potential bias from missing data.

The secondary outcome of time to institutionalization will be analyzed using standard stratified log-rank and Mantel-Haenszel tests, as used by the Early Breast Cancer Trialists' Collaborative Group study [42], and results presented as odds ratio plots. Any exploratory analyses here will use multivariate logistic and proportional hazards regression. Excessive subgroup analyses can give rise to misleading results and therefore all subgroup investigations will be interpreted cautiously.

#### Health Economic Evaluation

Service utilisation patterns, carer inputs and all associated costs will be calculated for each patient, based on data collected using a modified version of the CSRI, completed by a family carer or professional carer. Unit costs to reflect long-run marginal opportunity costs will be attached using national figures where available. Each cost-effectiveness analysis will be conducted from the perspective of (a) the NHS and social services, and (b) society. The BADLS, DEMQOL and a utility measure generated from the EQ-5D will be used in turn in a series of cost-effectiveness analyses, the last of these to generate Quality Adjusted Life Year (QALY) measures (with societal weights). The associations between EQ-5D, DEMQOL, SMMSE and BADLS scores, and changes therein, will also be examined, given uncertainty about the validity of EQ-5D measures as QALY generators within this population. Cost-effectiveness acceptability curves will be plotted using bootstrap analyses to locate the findings of the economic evaluation in their wider decision-making context. Sensitivity analyses will also examine the consequences of key assumptions in the cost-effectiveness analysis. In addition, a mathematical model, developed from the AD2000 database and using BADLS and NPI data, will be used to estimate risks of institutionalization in treatment groups over four years.

### Ethical considerations

The protocol has been approved by the Scotland A Research Ethics Committee, the MHRA and received local site R&D approval. The trial will be conducted in compliance with the European Union Clinical Trials Directive (2001/20/EC), the associated UK Medicines for Human Use (Clinical Trials) Regulations (2004) and Medicines for Human Use (Clinical Trials) Amendment Regulations 2006, the Data Protection Act (1998), the principles of ICH GCP guidelines (CPMP/ICH/135/95), the principles of the Declaration of Helsinki (1996) and other requirements as appropriate.

An Independent Data Monitoring Committee (IDMC) will monitor the progress of the trial including: recruitment, protocol adherence, serious adverse events and side effects of treatment as well as the difference between the trial treatments on the primary outcome measures. The IDMC will produce a report to the Trial Steering Committee (TSC) after every meeting and can recommend premature closure of the trial following clear evidence of benefit or harm in accordance with the IDMC charter.

The main ethical issue here is that the severity of cognitive impairment may significantly interfere with the individual patient's ability to give fully informed consent. With patients entering this study having SMMSE scores of between five and thirteen, the majority with this degree of dementia will lack the necessary mental capacity to give fully informed consent.

However, the aims of the study mean it is vital that a representative patient group is randomized including those that lack capacity. The DOMINO-AD study involves minimal risk to the patient and offers the potential of significant clinical benefit, so it is ethically permissible to randomize patients whose capacity is impaired. Approaches to and legislation relating to obtaining patients' agreement to participate in the study in this situation were described earlier.

## Discussion

There is considerable debate about the clinical and cost effectiveness of anti-dementia drugs. DOMINO-AD seeks to provide clear evidence on the best treatment strategies at a particularly important clinical transition point from moderate to severe AD. The design of the study, with multiple centres, a double-blind placebo controlled design, and central randomization, maximises recruitment opportunities and minimises the risk of selection or allocation bias. The results of existing trials have been influential in determining policy on prescribing for AD, but evidence for those people deteriorating to this transition point is lacking. This condition is certain to become more prevalent in ageing populations and therefore the decision on continuing treatment in people showing deterioration will be faced far more frequently in the future. The results of this study will make a substantial contribution to clinical decision-making in a situation currently characterised by uncertainty.

## Competing interests

Many of the investigators have received support from pharmaceutical companies, for example, to attend conferences, for giving lectures, for the provision of consultancy, for the conduct of research, or for the development of research infrastructure. Many investigators had no competing interests and the list of investigators with competing interests, and the details which they declared, is as follows:-

Professor Clive Ballard, King's College:

• Received honoraria from Novartis, Pfizer, Shire, Lundbeck, Myriad, Janssen-Cilag, Astra Zeneca and Servier pharmaceutical companies and research grants from Novartis, Lundbeck, Astra-Zeneca and Janssen-Cilag pharmaceuticals;

Professor Sube Banerjee, London, Maudsley:

• Development of the DEMQOL system;

• Received speaker and consultancy fees from all companies involved in making anti-dementia medication and has had an educational grant from Pfizer;

• Has worked for Department of Health;

Dr Peter Bentham, Birmingham:

• Is a paid consultant to Tau Therapeutics Pte Ltd;

Professor Alistair Burns, Manchester:

• Received research funding and honoraria and expenses for consultancy work from companies involved in the manufacturing and marketing of drugs for dementia – Eisai, Pfizer, Shire, Baxter, Janssen-Cilag. Does occasional lectures for companies hosting meetings on behalf of these industries – Pharam-Ed and Phase-V;

• Gets paid expenses from the Alzheimer's Society in the UK and Alzheimer's Australia (for lectures in 2008);

• Received an honorarium from John Wiley for role as editor of the International Journal of Geriatric Psychiatry and receive honoraria from a number of publishers for books written and edited;

Assoc. Professor Rob Jones, Nottingham:

• Has received educational travel and expenses support for a conference attendance from Pfizer and on another occasion from Boots;

• Has spoken at and/or organised educational events which have received educational funding support from companies with products in the field of Alzheimer's disease and related conditions;

Professor Roy Jones, Bath:

• His research institute has received grant support, consulting fees and honoraria from companies with products in the field of Alzheimer's disease and related conditions;

• The Research Institute for Care of the Elderly has recently completed the development of a new research building which has been funded as a result of donations to a major capital appeal. A number of pharmaceutical companies including Lundbeck, Merz, Eisai and Pfizer working in the field of Alzheimer's disease and dementia have contributed to this appeal;

Professor James Lindsay, Leicester:

• Has received speaker and consultancy fees from companies involved in the manufacturing and marketing of drugs for dementia – Eisai, Pfizer, Shire, Janssen-Cilag;

Professor John O'Brien, Newcastle:

• Received honoraria from Pfizer, Shire, Lundbeck, Janssen-Cilag and GE Healthcare, and provided consultancy to GE Healthcare, Servier and Bayer.

Dr Peter Passmore, Belfast:

• Has been a member of speaker bureaus in international meetings and conferences for Eisai, Janssen-Cilag, Lundbeck, Novartis, Pfizer and national meetings in UK for Bayer, Bristol-Myers Squibb, Astra Zeneca, Sanofi-Synthelabo, Merck Sharp & Dohme, Eisai, Shire, Novartis, Pfizer;

• Has been an advisor internationally for Bayer, Bristol-Myers Squibb, Eisai, Janssen-Cilag, Novartis, Pfizer, Sanofi-Synthelabo, and nationally in the UK for Bayer, Bristol-Myers Squibb, Astra Zeneca, Sanofi-Synthelabo, Merck Sharp & Dohme, Eisai, Shire, Lundbeck, Novartis, Pfizer;

Dr Bart Sheehan, Warwick:

• Has received an educational travel grant and conference expenses from Janssen-Cilag.

## Authors' contributions

RH conceived the study, assembled the group of investigators and co-investigators and, with them, developed and finalised the protocol. All of the above have set up the trial sites and made them ready to perform the trial. EJ, PP, RG and TJ provided statistical advice in the design of the study and its on-going evolution. RB assisted with psychometric aspects of the trial's development and MK and JR assisted with health economic aspects of the trial's development. JA, CO and MG managed the trial data management and co-ordinated development of the trial. RGJ, BS, PP and EJ particularly developed this publication describing the trial protocol. All the authors read and approved the final manuscript.
